# Supplementary material for: Distinct Localization of Mature HGF from its Precursor Form in Developing and Repairing the Stomach
Source: Int J Mol Sci. 2019 Jun 17;20(12):2955. doi: 10.3390/ijms20122955 (PMC6628191; doi:10.3390/ijms20122955)
Supplement: Supplementary file 1 [file ijms-20-02955-s001.pdf]

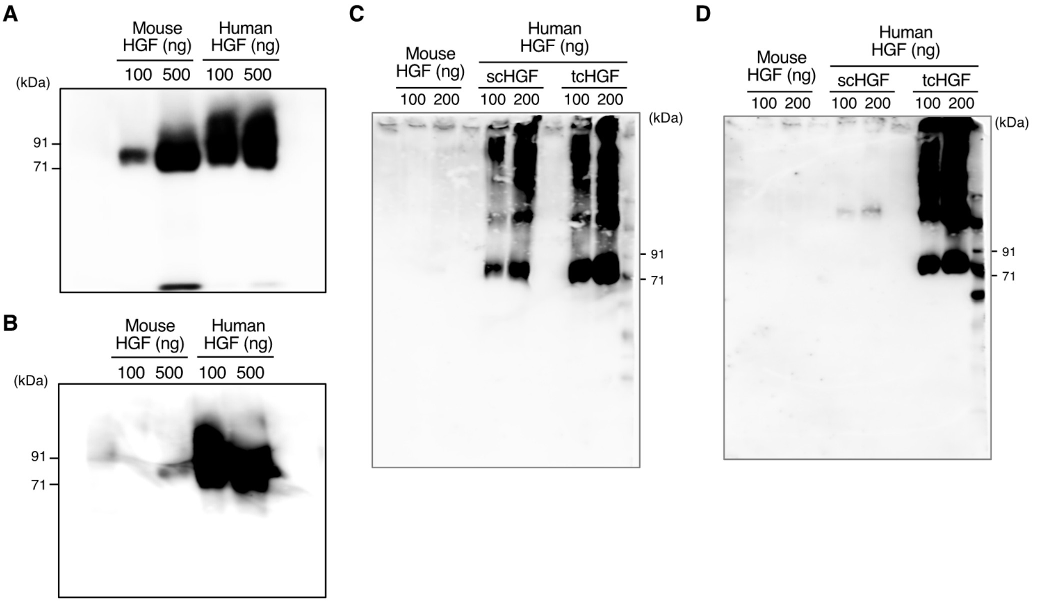

Figure S1. Original western blot images for Figure 1. Images in Figure 1A were cropped from (A) and (B). Images in Figure 1B were cropped from (C) and (D).

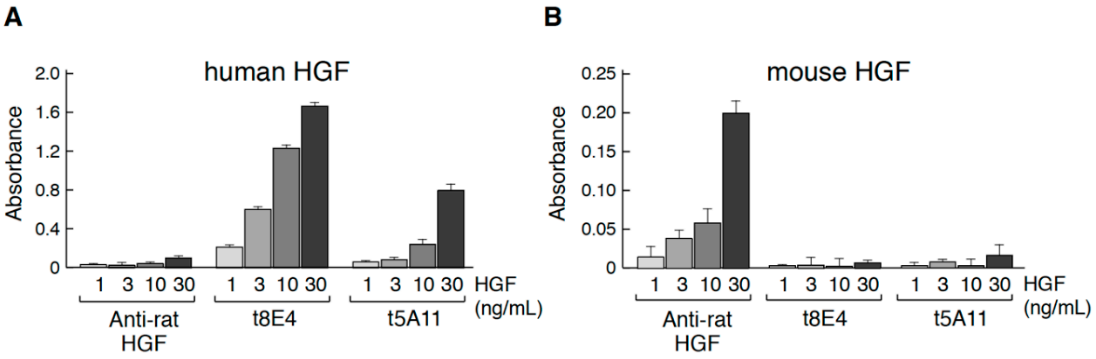

Figure S2. Specificity of t5A11 and t8E4 anti-human HGF antibodies. (A) Specificity to human HGF. (B) Specificity to mouse HGF. Specificity of antibodies was measured by ELISA. Wells in a 96-well plate were coated with 2 μg/mL antibody overnight at 4℃. Wells were washed with 0.05% Tween-20 in PBS (PBS-T), blocked with 3% (w/v) bovine serum albumin in PBS-T, and incubated with HGF for 1 h. After washing with PBS-T, wells were incubated with 2 μg/mL biotinylated anti-human or rat HGF IgG for 1 h and HRP-conjugated streptavidin for 30 min. The enzyme reaction was performed using *o*-phenylenediamine and H_2O_2 as substrates. The reaction was analyzed by measuring absorbance at 490 nm. Anti-rat HGF polyclonal antibody, which is selectively reactive to mouse and rat HGF but not human HGF, was used as positive control as described in the following: Xu, Q.; Nakayama, M.; Suzuki, Y.; Sakai, K.; Nakamura, T.; Sakai, Y.; Matsumoto, K. Suppression of acute hepatic injury by a synthetic prostacyclin agonist through hepatocyte growth factor expression. *Am. J. Physiol.* **2012**, 302, G420–G429, doi:10.1152/ajpgi.00216.2011.

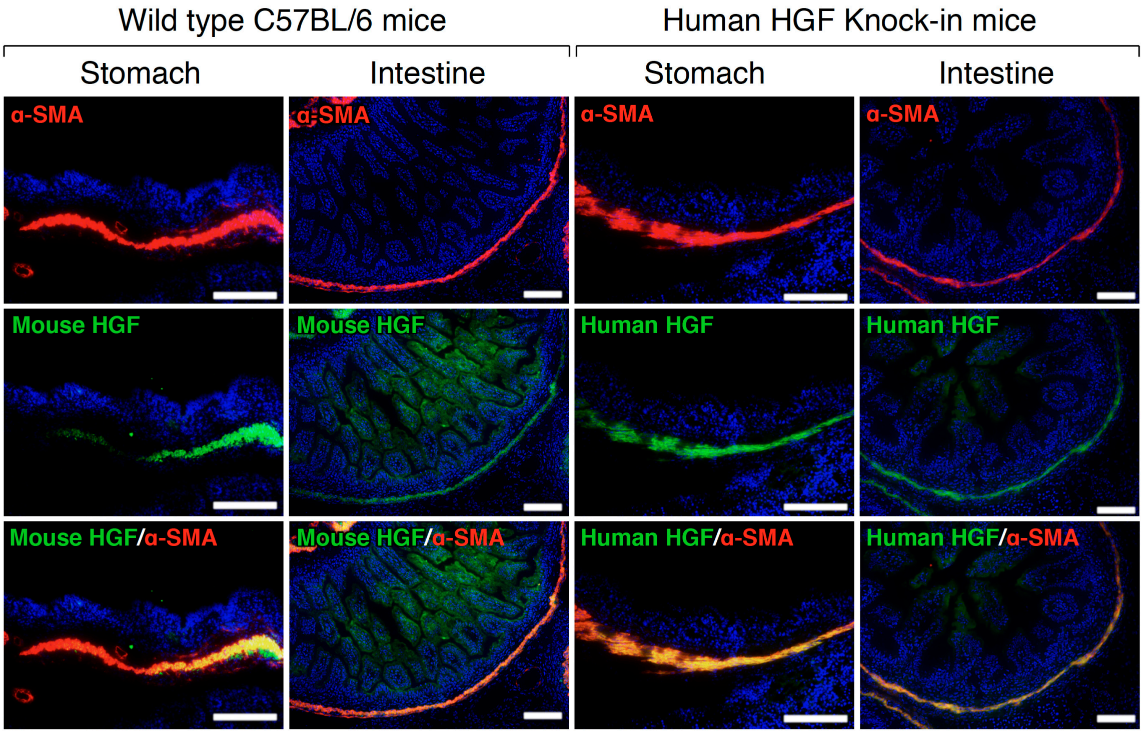

Figure S3. Co-localization of HGF and α-SMA in the developing stomach and intestine of wild-type C57BL/6 and hHGF-ki mice. Double immunofluorescence staining was performed using anti-mouse HGF antibody, t5A11 anti-human HGF antibody or anti-α-SMA antibody. Similar immunofluorescence localization patterns were obtained in sections from two different mice. Tissues were obtained from day 16.5 embryos from wild-type C57BL/6 or human HGF knock-in mice. Scale bars represent 200 μm.

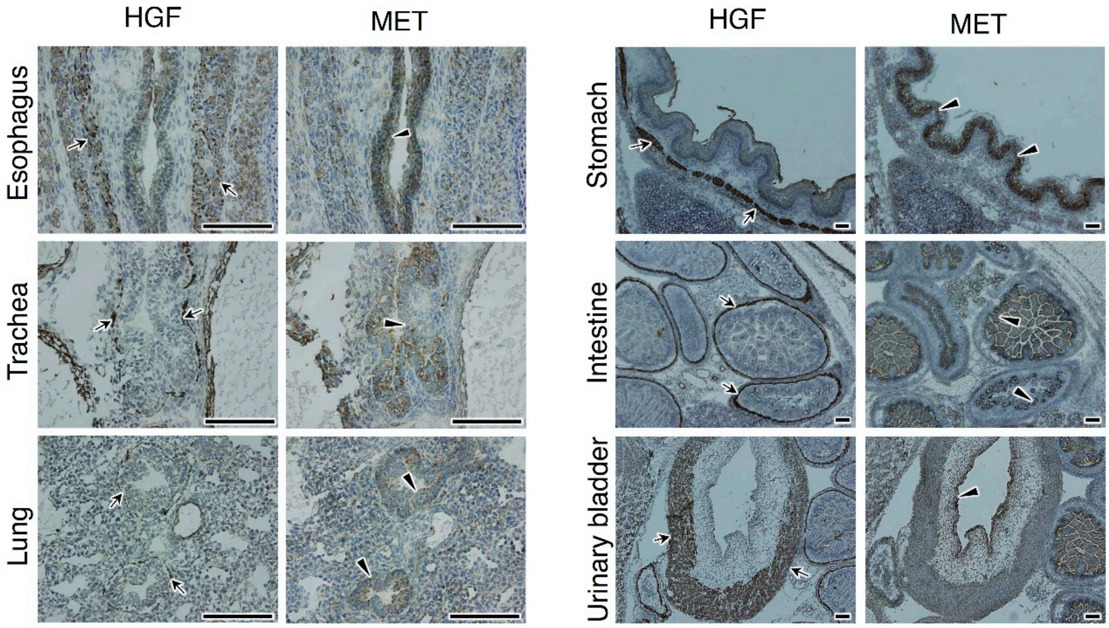

Figure S4. Localizations of HGF and MET receptor in several organs in the mouse embryo. Immunohistochemistry was performed using anti-human HGF monoclonal antibody (t5A11) or anti-MET antibody. Black arrows and arrowhead indicate the localizations of HGF and MET, respectively. Black arrows indicate that HGF was mainly localized in smooth muscle cells. Black arrowheads indicate that MET was mainly localized in epithelial cells. Similar immunohistochemical localization patterns were obtained in sections from two different mice. Tissues were obtained from day 16.5 embryos of hHGF-ki mice. Scale bars represent 200 μm.

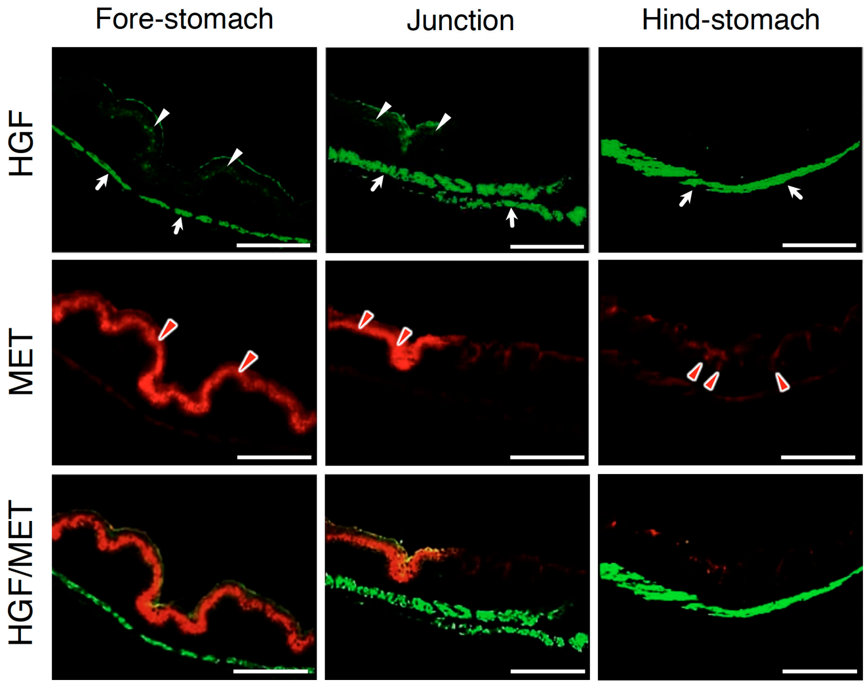

Figure S5. Localizations of HGF and MET receptors in the developing stomach. Immunofluorescent staining was performed using t5A11 anti-human HGF antibody or anti-MET antibody. White arrows and arrowheads indicate HGF localized in smooth muscle cells and epithelial cells, respectively. Red arrowheads indicate MET expression in epithelial cells. Similar localization patterns were obtained in sections from two different mice. Tissues were obtained from day 16.5 embryos of hHGF-ki mice. Scale bars represent 200 µm.

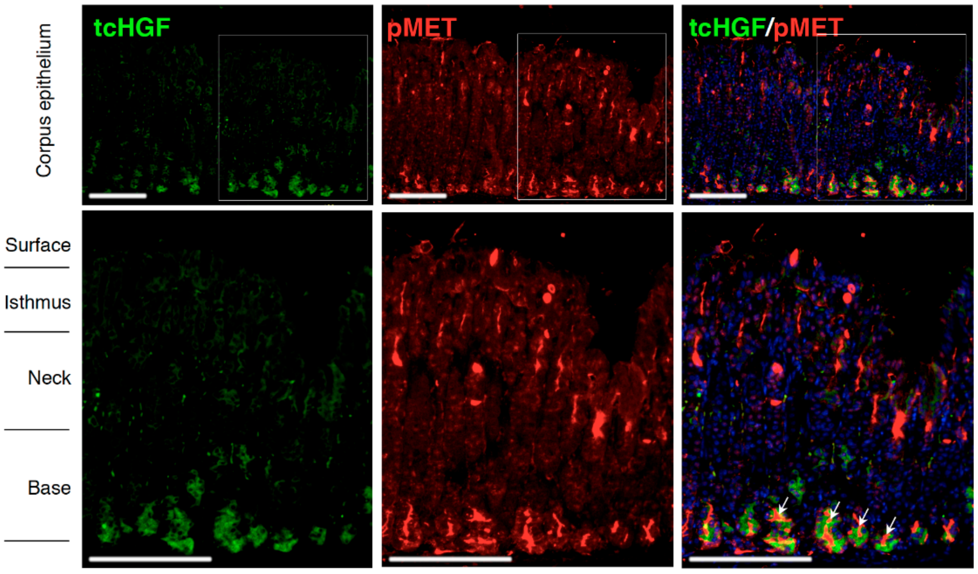

Figure S6. Localizations of tcHGF and pMET in the adult stomach. tcHGF and pMET were detected by double immunofluorescence staining. White arrows indicate co-localization of tcHGF and pMET in glandular base of the adult stomach. The images in the lower panel are magnified images of the boxed areas in the upper panel. Tissues were obtained from hHGF-ki mice. Scale bars represent 200 µm.

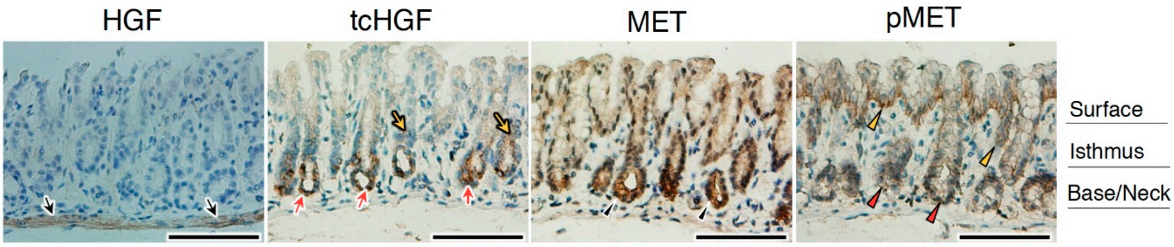

Figure S7. Localizations of HGF, tcHGF, MET, and pMET in the antral epithelium of the adult stomach. Immunohistochemical staining was performed using t5A11 (for scHGF and tcHGF), t8E4 (for tcHGF), anti-MET, or anti-phospho-MET antibody. Black arrows, HGF in smooth muscle cells; red arrows, tcHGF in the base region; yellow arrows, tcHGF in the neck region; black arrowheads, MET in the glandular base region; red arrowheads, pMET in the base region; yellow arrowheads, pMET in the isthmus and/or surface regions. Similar localization patterns were obtained in sections from different two mice. Tissues were obtained from hHGF-ki mice. Scale bars represent 200 μm.

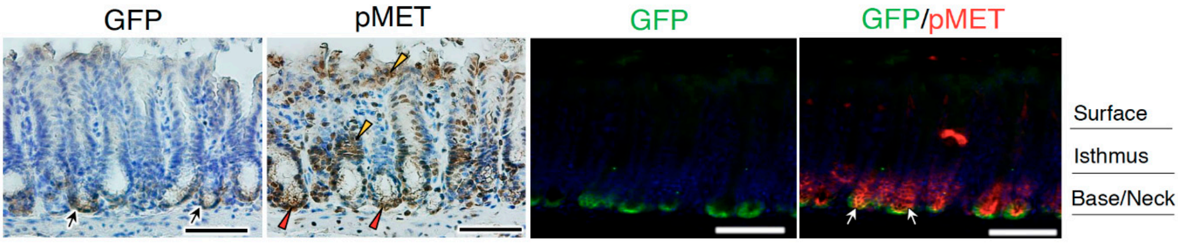

Figure S8. Localizations of Lgr5[+] stem cells and pMET in the antrum of adult stomach. Immunohistochemical and double immunofluorescence staining for GFP and pMET. Black arrows, Lgr5-driven GFP; red arrowheads, pMET in the glandular base region; yellow arrowheads, pMET in the isthmus and/or surface regions. White arrows indicate co-localization of pMET and GFP. Tissues were obtained from Lgr5-DTR-EGFP mice. Scale bars represent 200 μm.

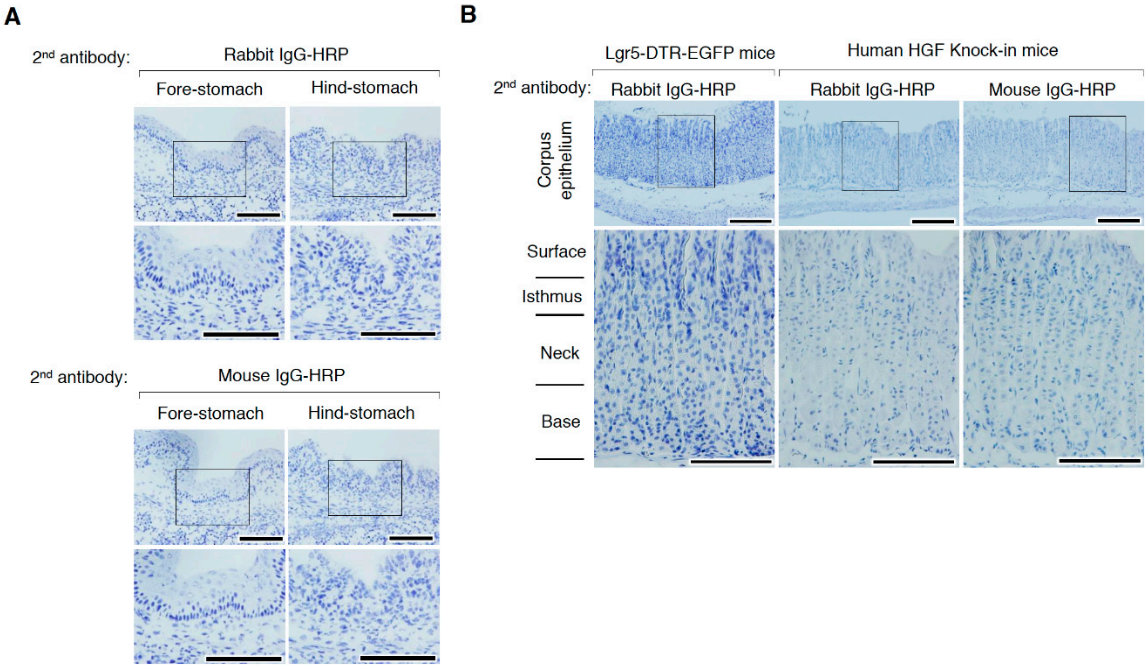

Figure S9. The negative controls for immunohistochemical staining**.** In A, tissues were obtained from day 16.5 embryos. In B, tissues were obtained from Lgr5-DTR-EGFP mice and human HGF knock-in mice. Immunohistochemical staining was performed without using primary antibodies. HRP-labeled anti-rabbit IgG or anti-mouse IgG was used as the secondary antibody. The images in the lower panel are magnified images of the boxed areas in the upper panel. Scale bars represent 200 μm.
